# Supplementary material for: Effects of Sodium Selenite on Accumulations of Selenium and GABA, Phenolic Profiles, and Antioxidant Activity of Foxtail Millet During Germination
Source: Foods. 2024 Dec 4;13(23):3916. doi: 10.3390/foods13233916 (PMC11641185; doi:10.3390/foods13233916)
Supplement: Supplementary file 1 [file foods-13-03916-s001.zip › foods-3297659-supplementary.pdf]

Table S1. The individual compounds identified using UPLC-ESI-MS/MS in the free fraction of the Se-enriched foxtail millet sprout samples.

NO.	Retention time(min)	$[M\text{-}H]^-$ (m/z)	m/z of main fragments (relative intensity, %), MS/MS	UV λ max (nm)	Formula	Compound identified
1	2.64	164	72(30),91(50),103(100),147(15)	257	$C_9H_{11}NO_2$	Phenylalanine*
2	4.47	203	74(30),116(100),142(30)	279	$C_{11}H_{12}N_2O_2$	Tryptophan*
3	4.90	137	93(100),108(30)	290	$C_7H_5O_3$	Protocatechuic aldehyde*
4	5.66	137	93(100)	297	$C_7H_5O_3$	p-hydroxybenzoic acid*
5	5.85	337	119(100),163(50),173(20),191(25)	308	$C_{16}H_{18}O_8$	3-p-coumaroylquinic acid
6	7.48	121	92(80),121(100)	318	$C_7H_5O_2$	p-hydroxybenzaldehyde
7	8.41	321	119(100),138(10),233(15)	292	$C_{19}H_{17}N_2O_3$	N-(p-coumaroyl) serotonin
8	10.98	351	147(20),175(30),193(15),231(90),249(60),351(15)	316	$C_{20}H_{20}N_2O_4$	N-feruloylserotonin
9	12.45	337	93(10),163(20),173(100),191(10)	311	$C_{16}H_{18}O_8$	4-p-coumaroylquinic acid
10	12.96	163	119(100)	309	$C_9H_7O_3$	trans-p-coumaric acid*
11	14.12	367	93(10),173(100),193(20)	325	$C_{17}H_{20}O_9$	Feruloylquinic acid
12	14.39	237	59(25),89(25),119(50),145(100)	308	$C_{12}H_{14}O_5$	1-O-p-coumaroylglycerol
13	14.88	593	353(5),383(15),473(10),503(15),593(100)	344	$C_{12}H_{14}O_5$	Apigenin-C-dihexoside
14	14.98	193	134(100)	320	$C_{10}H_9O_4$	trans-ferulic acid*
15	15.58	307	60(50),104(100),145(70)	289	$C_{16}H_{24}N_3O_2$	N′-caffeoylspermidine
16	15.72	563	353(10),443(15),473(10),563(100)	321	$C_{26}H_{27}O_{14}$	Apigenin-C-pentosyl-C-hexoside
17	16.13	436	119(55),142(10),274(10),316(100),436(50)	274	$C_{25}H_{30}N_3O_4$	N′, N″-di-p-coumaroylspermidine
18	16.13	563	383(10),443(15),473(20),503(15),563(100)	273	$C_{26}H_{27}O_{14}$	Apigenin-C-pentosyl-C-hexoside
19	16.38	466	119(50),145(90),175(20),326(100)	293	$C_{26}H_{32}N_3O_5$	N′-p-coumaroyl-N″-feruloylspermidine
20	16.61	496	135(85),149(30),346(100)	271	$C_{27}H_{34}N_3O_6$	N′,N″-diferuloylspermine
21	17.00	563	353(10),443(15),473(10),563(100)	321	$C_{26}H_{27}O_{14}$	Apigenin-C-pentosyl-C-hexoside
22	20.10	271	107(30),119(60),151(100)	324	$C_{15}H_{12}O_5$	Naringenin

23	20.25	609	327(10),357(15),429(20),489(20),609(100)	329	$C_{27}H_{29}O_{16}$	Kaempferol-C,O-dihexoside
24	21.09	413	119(20),145(25),163(90),193(100),267(15),398(40)	317	$C_{22}H_{22}O_8$	1-O-p-coumaroyl-3-O-feruloylglycerol
25	21.44	329	227(10),271(10),285(5),299(30),314(100)	317	$C_{17}H_{14}O_7$	3,7-dimethylquercetin

* The identification of the compound was confirmed by the authentic standard

Table S2. The individual compounds identified using UPLC-ESI-MS/MS in the bound fraction of the Se-enriched foxtail millet sprout samples.

NO.	Retention time(min)	$[M\text{-}H]^-$ (m/z)	m/z of main fragments (relative intensity, %), MS/MS	UV λ max (nm)	Formula	Compound identified
1	3.20	153		261	$C_7H_5O_4$	Protocatechuic acid*
2	5.17	137		277	$C_7H_5O_3$	Protocatechuic aldehyde*
3	5.93	137		255	$C_7H_5O_3$	p-hydroxybenzoic acid*
4	7.54	121	92(100)	220/284	$C_7H_5O_2$	p-hydroxybenzaldehyde*
5	8.24	167	63(20),80(100),108(95),152(50)	260/292	$C_8H_7O_4$	Vanillic acid*
6	10.55	151	153(40),182(60)	278	$C_8H_8O_3$	Syringic acid*
7	13.08	163	93(10),119(100)	226/309	$C_9H_7O_3$	trans-p-coumaric acid*
8	14.42	163	93(10),119(100)	296	$C_9H_7O_3$	cis-p-coumaric acid
9	15.09	193	134(100),178(10)	322	$C_{10}H_9O_4$	trans-ferulic acid*
10	15.71	385	108(30),123(40),173(50),217(40), 267(60),282(100),326(65),341(30)	329	$C_{20}H_{17}O_8$	8,8′-aryltetralin-DFA
11	16.07	193	134(100),178(10)	312	$C_{10}H_9O_4$	cis-ferulic acid
12	16.36	389	134(25),165(100),195(80),282(50)327(40)	323	$C_{20}H_{21}O_8$	Dihydroferulic acid dimer
13	16.64	385	267(20),282(70),326(75)	322	$C_{20}H_{17}O_8$	8,8′-DFA
14	17.93	387	123(15),134(10),193(100)	273	$C_{20}H_{19}O_8$	Ferulic truxilic acid
15	18.31	385	103(15),265(25),282(100),326(40),341(100), 385(75)	280	$C_{20}H_{17}O_8$	8-5′-DFA
16	18.55	385	235(20),267(80),282(100),326(70),341(20)	323	$C_{20}H_{17}O_8$	5-5′-DFA
17	18.55	577	148(30),163(30),193(20),394(25),445(10),489(20)	323	$C_{30}H_{25}O_{12}$	TFA
18	19.12	385	134(10),149(15),193(100),298(20)	325	$C_{20}H_{17}O_8$	Trans-trans-8-O-4′–DFA
19	19.43	385	123(15),195(15),267(100),282(60),326(50),341(15)	323	$C_{20}H_{17}O_8$	Trans-cis-8-O-4′–DFA
20	20.40	577	163(15),193(20),341(100),385(60),445(10),522(40)	285/319	$C_{30}H_{25}O_{12}$	TFA
21	21.53	329	227(10),271(10),285(5),299(50),314(100)	317	$C_{17}H_{14}O_7$	3,7-dimethylquercetin

* The identification of the compound was confirmed by the authentic standard. DFA: ferulic acid dimer. TFA: ferulic acid trimer.

Table S3. Effect of Na2SeO3 treatments on individual free phenolic compounds contents (mg/kg DW) of foxtail millet sprouts during germination process.

Phenolic compounds	Germination day											
	G1			G2			G3			G4		
**Free**	Control	Na_2SeO_3 (soaking)	Na_2SeO_3 (spraying)	Control	Na_2SeO_3 (soaking)	Na_2SeO_3 (sprayin g)	Control	Na_2SeO_3 (soaking)	Na_2SeO_3 (spraying)	Control	Na_2SeO_3 (soaking)	Na_2SeO_3 (spraying)
Protocatechuic aldehyde	0.71±0.02 Db	1.44±0.02 Ca	1.35±0.01 Ca	0.90±0.03 Cb	1.08±0.02 Da	0.72±0.02 Dc	1.53±0.06 Bc	3.38±0.06 Ba	1.94±0.03 Bb	2.71±0.03 Ab	4.75±0.07 Aa	2.82±0.04 Ab
p-hydroxybenzoic acid	0.85±0.01 Da	0.91±0.02 Da	0.89±0.02 Ca	1.25±0.01 Cb	1.73±0.03 Ca	0.85±0.01 Cc	2.85±0.06 Bc	8.16±0.12 Ba	4.60±0.12 Ab	5.26±0.08 Ab	12.16±0.1 6Aa	4.00±0.07 Bc
3-p-coumaroylquinic acid	1.42±0.01 Ca	1.55±0.01 Ca	1.56±0.01 Ca	1.54±0.01 Ca	1.75±0.01 Ca	1.28±0.02 Db	2.98±0.05 Bc	7.22±0.04 Ba	4.38±0.08 Ab	4.86±0.07 Ab	10.43±0.1 4Aa	3.94±0.05 Bc
p-hydroxybenzaldehyd e	0.58±0.04 Cc	0.98±0.01 Da	0.84±0.01 Cb	2.53±0.04 Ab	2.98±0.04 Aa	2.11±0.02 Ac	0.55±0.01 Cc	1.06±0.01 Ca	0.69±0.01 Db	0.89±0.01 Bc	1.63±0.01 Ba	1.14±0.02 Bb
N-(p-coumaroyl) serotonin	n.d.	n.d.	n.d.	2.22±0.01 Ca	2.59±0.07 Ca	1.98±0.01 Ca	5.14±0.11 Bc	12.32±0.1 7Ba	7.26±0.12 Bb	8.71±0.11 Ab	17.61±1.1 5Aa	8.96±0.27 Ab
N-feruloylserotonin	n.d.	n.d.	n.d.	n.d.	4.30±0.05 Ca	4.29±0.08 Ba	6.20±0.06 Ab	6.21±0.05 Bb	7.45±0.16 Aa	6.44±0.11 Ab	6.70±0.14 Aab	7.04±0.24 Aa
4-p-coumaroylquinic acid	1.84±0.01 Db	2.44±0.08 Da	2.18±0.01 Dab	4.88±0.08 Ca	4.70±0.08 Ca	4.78±0.11 Ca	10.84±0.14 Ac	12.77±0.0 6Ab	14.02±0.2 4Aa	10.34±0.1 9Bb	11.02±0.1 3Ba	11.26±0.1 3Ba
trans-p-coumaric acid	1.53±0.01 Ca	1.57±0.02 Ca	1.38±0.01 Db	2.01±0.01 Aa	1.51±0.02 Cb	1.46±0.01 Cb	1.67±0.02 Bb	1.80±0.03 Ba	1.72±0.01 Bb	2.01±0.01 Aa	1.94±0.01 Aa	1.97±0.02 Aa
Feruloylquinic acid	3.77±0.03 Cb	5.89±0.06 Da	5.18±0.06 Da	10.00±0.1 7Ba	9.85±0.16 Ca	9.86±0.25 Ca	14.92±0.69 Ab	17.84±0.5 2Aa	18.59±0.7 9Aa	11.60±0.4 0Ba	12.61±0.3 9Ba	11.78±0.3 8Ba
1-O-p-coumaroylglycerol	1.26±0.01 Ca	1.24±0.01 Ca	1.09±0.01 Cb	1.24±0.01 Ca	1.28±0.02 Ca	1.33±0.02 Ba	1.41±0.02 Bb	1.53±0.02 Ba	1.40±0.03 Bb	2.13±0.03 Ab	2.23±0.02 Aa	1.94±0.01 Ac

Apigenin-C-dihexoside	3.26±0.05 Ba	3.18±0.05 Ca	3.09±0.25 Ba	2.67±0.04 Ca	2.80±0.05 Ca	2.65±0.03 Ca	3.13±0.05 Bb	3.44±0.06 Ba	3.46±0.07 Aa	3.63±0.03 Aa	3.93±0.03 Aa	3.83±0.06 Aa
trans-ferulic acid	6.24±0.11 Ca	5.88±0.09 Ca	5.73±0.81 Ca	10.02±0.51Ba	8.54±0.42 Ba	9.35±0.41 Ba	10.62±0.51 Ba	10.96±0.71Aa	11.08±0.67Aa	12.02±0.26Aa	11.34±0.33Aa	11.01±0.64Aa
N′-caffeoylspermidine	0.29±0.01 Ba	0.30±0.02 Ba	0.30±0.01 Ba	0.30±0.01 Ba	0.30±0.02 Ba	0.28±0.00 Bb	0.31±0.01 Ba	0.31±0.01 Ba	0.32±0.02 Aa	0.33±0.03 Aa	0.34±0.02 Aa	0.33±0.01 Aa
Apigenin-C-pentosyl-C-hexoside	9.52±0.04 Aa	9.66±0.08 Aa	9.78±0.31 Aa	5.57±0.05 Ba	5.76±0.06 Ba	5.60±0.07 Ba	5.10±0.19 Ba	4.19±0.07 Cb	4.12±0.11 Cb	4.41±0.05 Ca	3.92±0.09 Dc	4.15±0.19 Cb
N′, N″-di-p-coumaroylspermidine	2.71±0.01 Ab	2.78±0.01 Ab	2.90±0.01 Aa	1.99±0.02 Ba	2.03±0.01 Ba	1.93±0.01 Ba	2.18±0.01 Aa	1.97±0.03 Bb	2.00±0.02 Bab	1.40±0.01 Ca	1.29±0.01 Cb	1.23±0.01 Cb
Apigenin-C-pentosyl-C-hexoside	12.48±0.08Aa	12.49±0.12Aa	12.99±0.09 Aa	11.42±0.08Aa	11.58±0.07Aa	9.95±0.05 Bb	9.16±0.07 Ba	6.55±0.2Bb	6.68±0.05 Cb	4.14±0.07 Ca	3.57±0.05 Cb	4.16±0.14 Da
N′-p-coumaroyl-N″-feruloylspermidine	9.80±0.05 Aa	10.07±0.04Aa	10.64±0.07 Aa	6.53±0.07 Cb	5.96±0.04 Bc	7.46±0.18 Ba	7.57±0.14 Ba	5.95±0.14 Bb	4.21±0.04 Bc	4.10±0.04 Da	3.62±0.04 Cab	3.38±0.08 Ca
N′,N″-diferuloylspermine	2.66±0.04 Ca	2.83±0.04 Ba	2.79±0.04 CDa	2.58±0.05 Ca	2.65±0.05 Ca	2.6±0.08 Da	3.33±0.04 Ba	3.02±0.04 Bb	3.39±0.04 Aa	3.88±0.07 Aa	3.25±0.05 Ab	2.85±0.03 BCc
Apigenin-C-pentosyl-C-hexoside	7.28±0.19 Aa	7.33±0.19 Aa	7.46±0.06 Aa	4.99±0.2Ba	5.03±0.18 Ba	5.12±0.06 Ba	4.70±0.09 Ba	4.21±0.03 Ca	4.30±0.08 Ca	3.46±0.03 Cb	3.58±0.05 Dab	3.82±0.11 Ca
Kaempferol-C,O-dihexoside	4.31±0.07 Aa	2.87±0.03 Ac	2.97±0.02 Ab	n.d.	n.d.	n.d.	n.d.	n.d.	n.d.	n.d.	n.d.	n.d.
1-O-p-coumaroyl-3-O-feruloylglycerol	9.18±0.05 Ba	8.73±0.17 Ca	8.94±0.18 Ba	7.38±0.15 Cb	7.25±0.09 Db	9.50±0.12 Ba	8.05±0.19 BCb	12.15±0.52Ba	8.66±0.09 Bb	11.24±0.6 Ac	14.55±0.36Ab	16.86±0.47Aa
3,7-dimethylquercetin	7.12±0.08 Ca	6.63±0.13 Ca	8.00±0.14 Ca	6.27±0.06 Cb	6.05±0.06 Cb	7.85±0.07 Ca	10.51±0.48 Bc	17.46±0.35Ba	15.36±0.72Bb	19.86±0.2 Ac	25.70±0.48Aa	23.81±0.47Ab

Different capital letters indicate significant differences at different germination days ($p<0.05$), while different lowercase letters indicate significant differences in different germination treatments ($p<0.05$)

n.d., not detected

DW, dry weight of sample

Results are expressed as mean ± SD

Table S4. Effect of Na2SeO3 treatments on individual bound phenolic compounds contents (mg/kg DW) of foxtail millet sprouts during germination process.

Phenolic compounds	Germination day											
	G1			G2			G3			G4		
**Bound**	Control	Na_2SeO_3 (soaking)	Na_2SeO_3 (spraying)	Control	Na_2SeO_3 (soaking)	Na_2SeO_3 (spraying)	Control	Na_2SeO_3 (soaking)	Na_2SeO_3 (spraying)	Control	Na_2SeO_3 (soaking)	Na_2SeO_3 (spraying)
Protocatechuic acid	3.07±0.06Aa	2.93±0.08Bb	2.94±0.03Cb	2.80±0.03Ba	2.39±0.13Cb	2.83±0.02Da	2.70±0.32Cc	3.59±0.04Aa	3.10±0.13Bb	3.14±0.14Ac	3.60±0.09Ab	5.79±0.06Aa
p-hydroxybenzaldehyde	8.96±0.11Ba	7.73±0.08Cb	7.35±0.21Cc	6.64±0.11Db	6.60±0.15Db	7.28±0.12Ca	7.94±0.37Cc	11.00±0.51Ba	8.60±0.21Bb	10.84±0.28Ac	13.00±0.50Ab	18.28±0.79Aa
Vanillic acid	6.14±0.10Ba	5.84±0.15Cb	6.04±0.17Ca	5.80±0.08Ca	5.21±0.19Db	5.79±0.13Da	5.37±0.04Dc	7.95±0.17Ba	6.67±0.08Bb	7.32±0.24Ac	8.43±0.15Ab	13.35±0.36Aa
Syringic acid	6.80±0.11Ba	5.73±0.21Db	5.20±0.14Cc	5.67±0.03Db	6.25±0.01Ca	5.14±0.11Cc	6.17±0.24Cc	10.11±0.39Ba	7.27±0.29Bb	9.34±0.09Ac	11.43±0.49Ab	12.60±0.17Aa
trans-p-coumaric acid	203.78±1.89Ba	176.24±1.69Cb	198.57±2.68Ba	162.88±2.75Cb	149.14±1.09Dc	190.24±0.84Ca	158.50±1.79Cc	237.17±1.21Ba	202.23±2.59Bb	251.60±1.84Ac	319.26±4.29Ab	496.66±3.28Aa
cis-p-coumaric acid	7.99±0.63Aa	6.47±0.53Cb	5.90±0.72Cc	5.79±0.13Db	5.30±0.31Dc	6.02±0.51Ba	6.08±0.53Cb	7.14±0.63Ba	5.75±0.23Dc	6.95±0.73Bc	7.49±0.67Ab	11.14±0.43Aa
trans-ferulic acid	475.76±3.78Dc	543.30±1.73Db	612.56±2.28Ca	525.45±2.38Cb	581.15±1.58Ca	577.62±3.55Da	727.39±1.91Bc	970.22±3.18Bb	1016.00±3.51Ba	1140.92±2.29Ac	1327.39±3.08Ab	1440.34±2.78Aa
cis-ferulic acid	51.10±0.76Da	46.93±1.32Cb	29.60±1.16Dc	57.00±1.56Ca	49.03±0.74Cb	33.87±0.68Cc	61.40±1.28Bb	71.00±0.76Ba	44.14±0.86Bc	79.99±1.05Aa	80.18±2.76Aa	51.71±1.58Ab
Dihydroferulic acid dimer	12.25±0.29Ba	9.78±0.21Cb	10.56±0.14Cb	9.12±0.41Cb	10.07±0.17Ca	10.84±0.19Ca	9.56±0.11Cc	14.38±0.26Ba	13.27±0.15Bb	15.09±0.36Ac	17.23±0.19Ab	29.58±0.26Aa
8,8′-DFA	9.20±0.19Db	8.95±0.16Db	10.52±0.23Da	10.29±0.24Cb	11.82±0.19Ca	11.77±0.18Ca	20.51±0.15Bc	26.84±0.37Ba	25.51±0.26Bb	31.54±0.30Ac	46.86±0.18Ab	53.72±0.33Aa

8-5′-DFA	6.85±0.16 Cb	6.47±0.13 Dc	7.37±0.24 Ca	6.97±0.09 Cb	7.79±0.23 Ca	7.01±0.12 Db	10.25±0.22Bc	14.93±0.28Ba	11.83±0.16 Bb	16.51±0.16 Ab	19.85±0.26 Aa	19.40±0.29 Aa
5-5′-DFA	12.19±0.08Dc	13.93±0.15 Db	19.07±0.11Ca	13.66±0.04Cc	19.71±0.07Ca	15.84±0.18Db	28.71±0.22Bc	36.67±0.13Bb	40.64±0.16 Ba	47.88±0.58 Ac	61.36±0.94 Aa	55.48±1.04 Ab
TFA	12.19±0.08Dc	13.93±0.15 Db	19.07±0.11Ca	13.66±0.04Cc	19.71±0.07Ca	15.84±0.18Db	28.71±0.22Bc	36.67±0.13Bb	40.64±0.16 Ba	47.88±0.58 Ac	61.36±0.94 Aa	55.48±1.04 Ab
Trans-trans-8-O-4′–DFA	19.82±0.75Cb	19.46±0.68 Db	21.52±0.86Ca	20.60±1.38Cb	23.44±0.68Ca	20.98±0.58Cb	33.36±0.77Bc	46.15±0.93Ba	39.21±1.28 Bb	53.84±1.78 Ac	67.92±1.11 Ab	82.80±2.38 Aa
Trans-cis-8-O-4′–DFA	16.31±0.58Da	16.66±0.42 Da	16.36±0.18Da	19.76±0.32Cab	21.26±0.33Ca	18.29±0.23Cb	35.06±0.51Bc	43.91±0.13Ba	40.89±0.58 Bb	62.32±0.88 Ac	75.34±0.76 Ab	84.78±0.58 Aa
TFA	3.87±0.07 Cb	4.69±0.08 Ca	3.43±0.08 Cb	4.00±0.26 Ca	4.07±0.10 Ca	3.13±0.07 Cb	5.48±0.10 Bb	9.96±0.75 Ba	5.70±0.04Bb	9.66±0.56Ac	13.41±0.29 Aa	11.58±0.77 Ab
3,7-Dimethylquercetin	8.22±0.13 Ba	5.78±0.12 Dc	7.49±0.09 Cb	6.09±0.12 Db	7.57±0.11 Ca	7.63±0.07 Ca	6.94±0.25 Cc	9.34±0.11 Ba	8.11±0.17Bb	9.96±0.13Ac	12.14±0.51 Ab	21.76±0.70 Aa

Different capital letters indicate significant differences at different germination days ($p<0.05$), while different lowercase letters indicate significant differences in different germination treatments ($p<0.05$)

DFA: ferulic acid dimer. TFA: ferulic acid trimer

DW, dry weight of sample

Results are expressed as mean ± SD

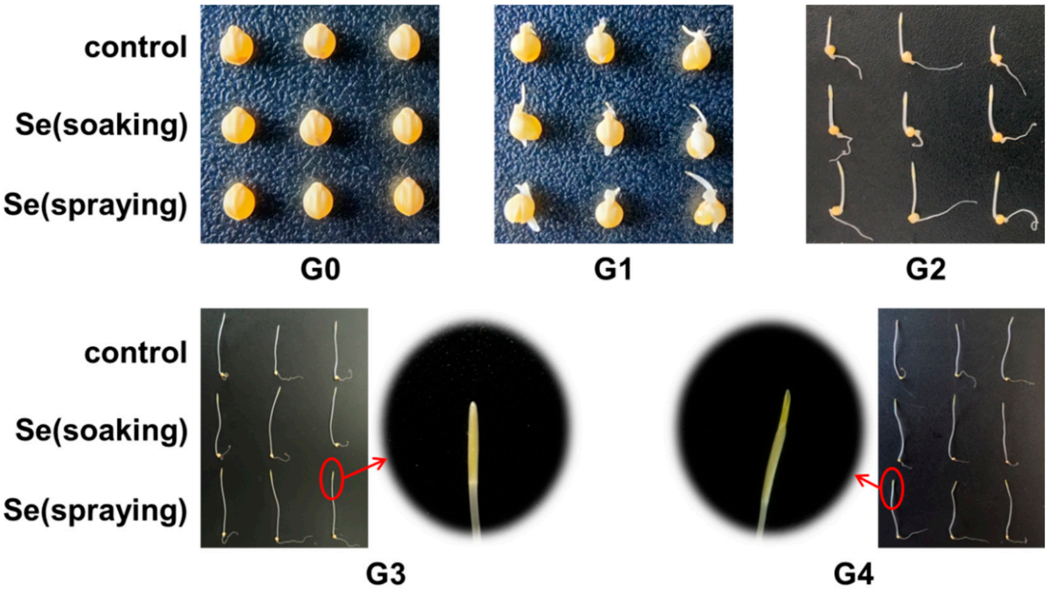

Figure S1. The changes in millet growth morphology under different germination treatments. Note: G0 represents the ungerminated raw seed; G1, G2, G3, and G4 represent foxtail millet sprouted for 0, 1, 2, 3, and 4 days, respectively. Se(soaking) represents the Na_2SeO_3 soaking treatment, and Se(spraying) represents the Na_2SeO_3 spraying treatment.

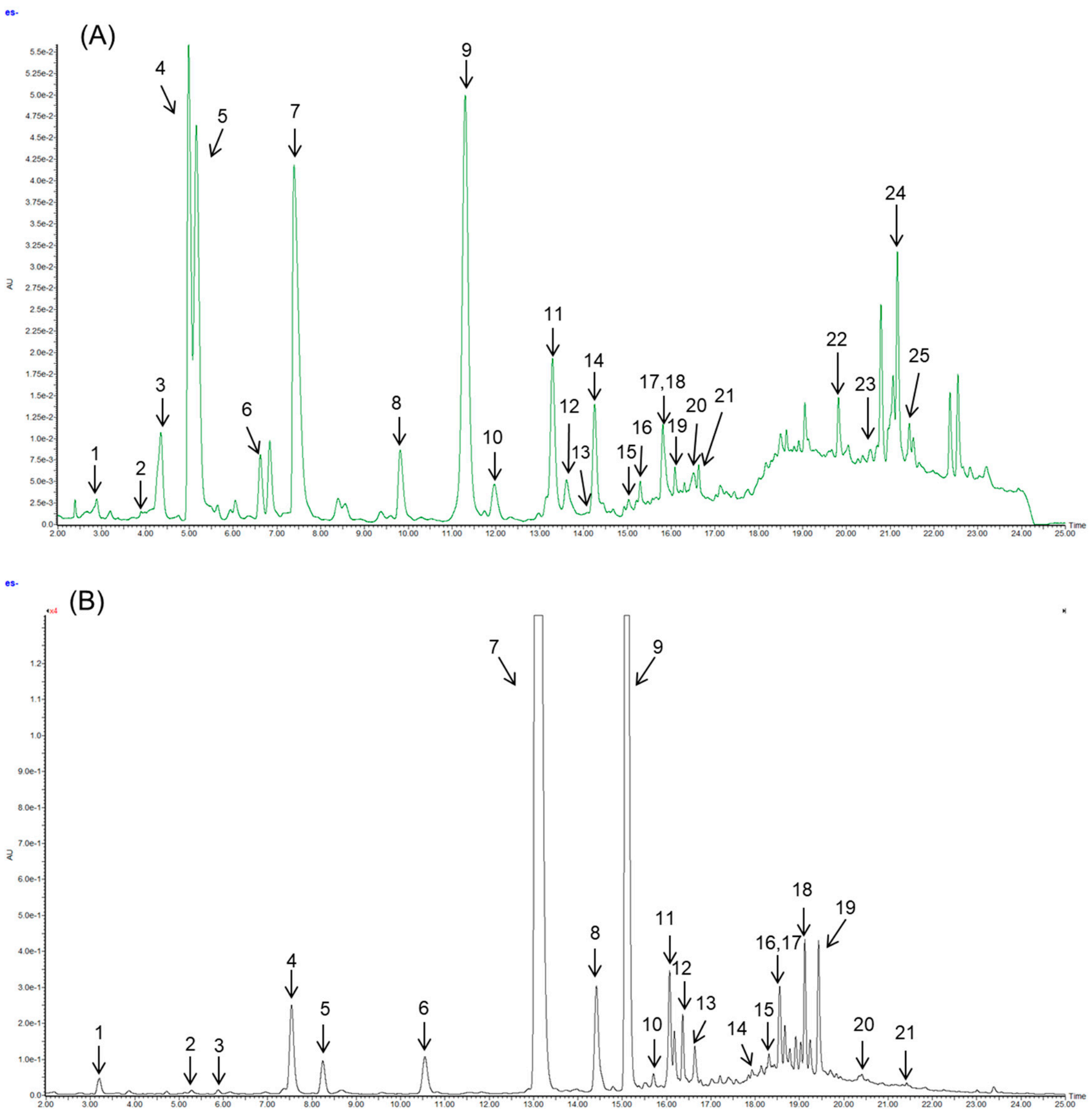

Figure S2. UPLC chromatograms of the free and bound phenolic extracts from Se-enriched foxtail millet sprout samples. (A) Free phenolic extract detection was set at a wavelength of 320 nm. (B) Bound phenolic extract detection was set at a wavelength of 280 nm. Note: The numbers in the figure represent the identified characteristic peaks.
